# A 50-m Forest Cover Map in Southeast Asia from ALOS/PALSAR and Its Application on Forest Fragmentation Assessment

**DOI:** 10.1371/journal.pone.0085801

**Published:** 2014-01-22

**Authors:** Jinwei Dong, Xiangming Xiao, Sage Sheldon, Chandrashekhar Biradar, Geli Zhang, Nguyen Dinh Duong, Manzul Hazarika, Ketut Wikantika, Wataru Takeuhci, Berrien Moore

**Affiliations:** 1 Department of Microbiology and Plant Biology, and Center for Spatial Analysis, University of Oklahoma, Norman, Oklahoma, United States of America; 2 International Center for Agricultural Research in Dry Areas, Amman, Jordan; 3 Institute of Geographic Sciences and Natural Resources Research, Chinese Academy of Sciences, Beijing, China; 4 Institute of Geography, Vietnam Academy of Science and Technology, Hanoi, Vietnam; 5 Geoinformatics Center, Asian Institute of Technology, Pathumthani, Thailand; 6 Center for Remote Sensing, Institute of Technology Bandung, Bandung, Indonesia; 7 Institute of Industrial Science, The University of Tokyo, Meguro, Tokyo, Japan; 8 College of Atmospheric and Geographic Sciences, University of Oklahoma, Norman, Oklahoma, United States of America; DOE Pacific Northwest National Laboratory, United States of America

## Abstract

Southeast Asia experienced higher rates of deforestation than other continents in the 1990s and still was a hotspot of forest change in the 2000s. Biodiversity conservation planning and accurate estimation of forest carbon fluxes and pools need more accurate information about forest area, spatial distribution and fragmentation. However, the recent forest maps of Southeast Asia were generated from optical images at spatial resolutions of several hundreds of meters, and they do not capture well the exceptionally complex and dynamic environments in Southeast Asia. The forest area estimates from those maps vary substantially, ranging from 1.73×10^6^ km^2^ (GlobCover) to 2.69×10^6^ km^2^ (MCD12Q1) in 2009; and their uncertainty is constrained by frequent cloud cover and coarse spatial resolution. Recently, cloud-free imagery from the Phased Array Type L-band Synthetic Aperture Radar (PALSAR) onboard the Advanced Land Observing Satellite (ALOS) became available. We used the PALSAR 50-m orthorectified mosaic imagery in 2009 to generate a forest cover map of Southeast Asia at 50-m spatial resolution. The validation, using ground-reference data collected from the Geo-Referenced Field Photo Library and high-resolution images in Google Earth, showed that our forest map has a reasonably high accuracy (producer's accuracy 86% and user's accuracy 93%). The PALSAR-based forest area estimates in 2009 are significantly correlated with those from GlobCover and MCD12Q1 at national and subnational scales but differ in some regions at the pixel scale due to different spatial resolutions, forest definitions, and algorithms. The resultant 50-m forest map was used to quantify forest fragmentation and it revealed substantial details of forest fragmentation. This new 50-m map of tropical forests could serve as a baseline map for forest resource inventory, deforestation monitoring, reducing emissions from deforestation and forest degradation (REDD+) implementation, and biodiversity.

## Introduction

Dramatic changes in forests, especially tropical forests, have significant impacts on regional climate, water and carbon cycles as well as biodiversity [1], [2]. Southeast Asia consists of 11 countries (Cambodia, Laos, Myanmar, Thailand, Vietnam, Malaysia, Brunei, Indonesia, Philippines, Singapore, and East Timor) and its population increased rapidly from approximately 359 million in 1980 to 593 million in 2010 [3]. To meet the rising demand for food, fiber, water, and housing, substantial land use and land cover changes, especially deforestation, have taken place in the region. As the third largest area of tropical rainforests in the world following the Amazon and Congo Basin [4], [5], Southeast Asia experienced more dramatic deforestation than any other continent in annual rate in the 1990s [6]. This region was also a hotspot of forest cover change from 2000 to 2010, e.g., Margono et al. [7]. For example, Indonesia and Myanmar were listed among the top ten countries with the largest annual net loss of forests while Vietnam had a large annual net gain of forest area from 2000 to 2010 [8]. National forestry policies differ between these countries, which further contributes to different forest change patterns and asymmetric forest transitions affected by the wood product trades among them [9]. Although government and scientists have made efforts to reinforce forest protection in Southeast Asia, forest conversion to plantations (e.g. oil palm) has continued to increase, which leads to fragmentation and affects biodiversity and carbon sequestration in peatlands [10]–[12]. To support regional sustainable development, including forest management, carbon emission estimation, habitat planning, and biodiversity conservation, it is critical that accurate and updated information on forest area, extent, fragmentation and change is developed [13], [14].

Several efforts have been carried out to map forest extent and change in Southeast Asia using optical remotely sensed data from the Advanced Very High Resolution Radiometer (AVHRR) [15]–[17], SPOT-Vegetation [4], [18], Moderate Resolution Imaging Spectroradiometer (MODIS) [14], [19], [20], and Medium Resolution Image Spectrometer (MERIS) [21]. However, the area estimates of forest cover in Southeast Asia from the above-mentioned studies differ substantially [4], [15]–[18], [22]. For example, the MODIS-based land cover product (MCD12Q1) estimated a forest area of 2.69×10^6^ km^2^ in Southeast Asia in 2009, the MERIS-based GlobCover land cover product estimated a forest area of 1.73×10^6^ km^2^ in 2009, and the FAO Forest Resources Assessment (FRA) 2010 reported a forest area of 2.14×10^6^ km^2^. All these three data products are widely used today for forest resource survey, climate simulation and biodiversity conservation [23]. The large discrepancy among these forest maps may be attributed to (1) fragmented forests or mixed pixels at moderate spatial resolutions (several hundreds of meters to 1-km), (2) frequent cloud cover [23]–[25], and (3) individual definitions and algorithms for forests. Although Landsat TM/ETM+ imagery (30-m spatial resolution) is available and has been widely used to map forests [14], [19], [22], [26], frequent cloud cover in the humid tropical zone makes it difficult to obtain cloud-free Landsat images over the entirety of Southeast Asia during a certain period. Consequently, the sample-based approach was used to select cloud-free Landsat images and map forest cover change [6], [14], [27]–[29]. This sampling approach does provide statistically-inferred information on forest cover change at national and continental scales, but forest management and decision-making for forest resource planning and biodiversity conservation requires location-specific, detailed, and updated annual maps of forests. Therefore, annual continental-scale forest maps of Southeast Asia at higher spatial resolutions (<100 m) are critical and urgently needed.

Synthetic aperture radar (SAR) with fine spatial resolution provides cloud-free imagery and is an alternate source for tropical forest mapping [30]–[32]. A long radar wavelength (e.g. L-band SAR) has an improved capability to delineate high-biomass forest than shorter wavelengths (e.g., C-band SAR) because of its greater penetration capability through the tree canopy [33]. Early in the 1990s, a single HH polarization dataset from the Japanese Earth Resources Satellite (JERS-1) was used for forest mapping (e.g. clear-cut) [34], [35]; however, continental forest maps based on JERS-1 imagery have not yet been developed. The Phased Array Type L-band Synthetic Aperture Radar (PALSAR), onboard the Advanced Land Observing Satellite (ALOS) launched by the Japan Aerospace Exploration Agency (JAXA) in January of 2006, provides an enhanced capacity for forest mapping and deforestation detection [31]–[33], [36]–[43]. JAXA has generated the first 10-m global forest/non-forest map by using ALOS/PALSAR data [44], [45], and the Woods Hole Research Center in Massachusetts, USA, is developing a pan-tropical forest cover map for subsequent deforestation and forest degradation monitoring by using ALOS/PALSAR data [46]. However, both of these are still unavailable to the public at this time.

The objective of this study was twofold: (1) to generate a map of tropical forest cover in Southeast Asia in 2009 at 50-m spatial resolution; and (2) to evaluate the fragmentation of forests in the region, based on the new and improved forest cover map. The forest map was generated by using the publicly available ALOS/PALSAR 50-m orthorectified mosaic imagery and the decision tree method reported in a previous study [47]. We evaluated the resultant map with validation sample data derived from the ground truth field photos at the Global Geo-referenced Field Photo Library (http://www.eomf.ou.edu/photos) and Google Earth. It was also compared to two regional/global land cover maps and to the FAO FRA 2010 statistics to supplement the validation. Based on this first continental-scale forest map at 50-m spatial resolution in Southeast Asia, forest fragmentation was investigated by using the fragmentation model presented by Riitters et al. [48], [49]. We aimed to better understand the fragmentation of forests in the region, which may help improve habitat planning and biodiversity conservation.

## Data and Methods

### PALSAR 50-m Orthorectified Mosaic Product and pre-processing

As part of the ALOS Kyoto and Carbon Initiative Project, JAXA released the PALSAR 50-m Orthorectified Mosaic Imagery Product in 2007, 2008, and 2009 for many parts of the world, including Southeast Asia. In this study we used the PALSAR 50-m Orthorectified Mosaic Product derived from images collected from June to October in 2009, and it has HH (horizontally transmitted and horizontally received) and HV (horizontally transmitted and vertically received) polarizations. The datasets are freely available to the public at the ALOS Kyoto and Carbon Initiative official website (http://www.eorc.jaxa.jp/ALOS/en/). The original PALSAR data with the observational mode of Fine Beam Dual (FBD) polarization has an off-nadir angle of 34.3 degrees, a range resolution of 14–88 m, and they have been geometrically rectified and mosaicked [50]–[52]. The Digital Number (DN) values (amplitude values) of these images were converted into the normalized radar cross section in decibel (σ*^0^*, with the unit of dB) according to the following formula [36],
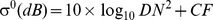
(1)where *DN* is the original digital number value in HH or HV polarization, and *CF* is the absolute calibration factor and equal to −83.

**Figure 1:**
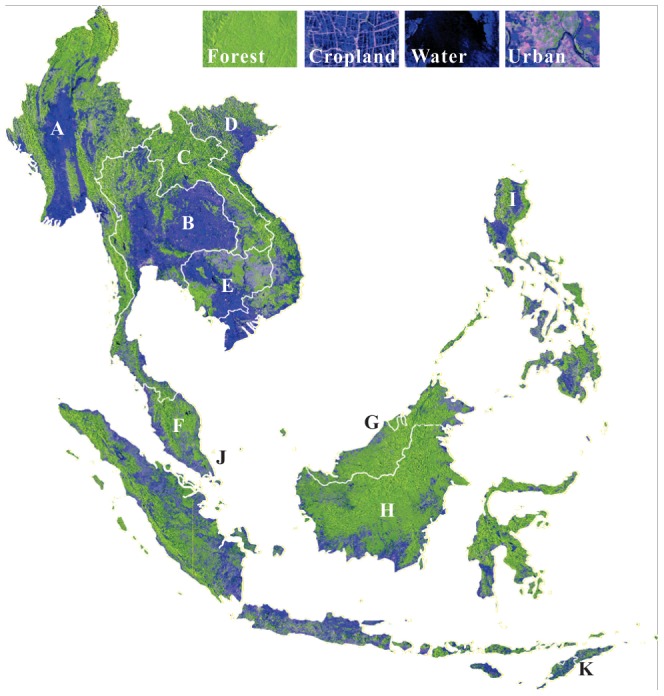
The false color composite of PALSAR 50-m orthorectified mosaic imagery (R/G/B  =  HH/HV/HH-HV) in Southeast Asia in 2009. Country names were labeled as Myanmar (A), Thailand (B), Laos (C), Vietnam (D), Cambodia (E), Malaysia (F), Brunei (G), Indonesia (H), Philippines (I), Singapore (J), and East Timor (K). The inset graphs show forest, cropland, water body, and built-up land, respectively. The PALSAR 50-m mosaic data was unavailable in the West Papua and Papua regions.

We generated two additional images from HH and HV images: (1) the band ratio (HH/HV) image and (2) the band difference (HH–HV) image. Both the difference image and band ratio image have proven valuable for land cover classification [53], [54] as they provide additional information of different land cover types. For example, the difference image was used to separate palm plantations and other trees [10]. The false color composite map (Fig. 1) shows the separability capability of the PALSAR image in four main land cover types.

### Ground-based Points of Interest (POIs) data

Geo-referenced field photos available at the Global Geo-Referenced Field Photo Library (see http://www.eomf.ou.edu/photos/, available to the public) at the University of Oklahoma (Xiao et al., 2011) were used as ground-based points of interest (POIs) data. The field photos and associated land cover information were provided by citizen scientists and researchers and archived at the Field Photo Library. We acquired the field photos only at unrestricted locations where no special permission was required and avoided the national park and other protected areas of land or sea related to wildlife. We confirm that our field studies did not involve endangered or protected species or provide specific location information for such.

**Figure 2:**
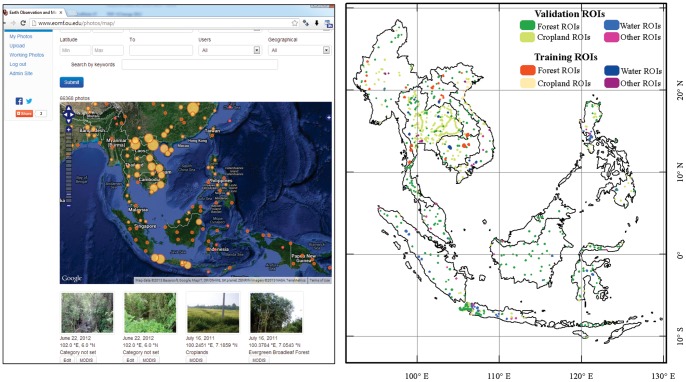
The spatial distribution of geo-referenced field photos in the study area, as hosted in (*A*) the Global Geo-referenced Field Photo Library. The circle size indicates the number of the field photos. The figure also shows the search options, selected photos with GPS locations, and the link to extract the MODIS time series data. More information can be found in the data portal (www.eomf.ou.edu/photos). (*B*) The Regions of Interest (ROIs) used for the algorithm training and results validation, which were acquired by referring to the field photos shown in Fig. 2*A* and Google Earth.

Researchers usually take photos to document their study sites and landscapes by using (a) GPS cameras, (b) smartphones, or (c) digital cameras plus handheld GPS receivers. The Field Photo Library is a community remote sensing and citizen science data portal for archiving, sharing, and exchanging these geo-tagged field photos and associated thematic databases of land cover types from the individual photos [55]. It hosts field photos contributed by our research team and other researchers around the world. Many field photos were interpreted and labeled by photo providers with the land cover types they represent, which results in a thematic database of land cover types, associated with the field photos. The geo-referenced field photos can be downloaded in different formats, compatible with Google Earth (kml or kmz), ArcGIS (shapefile), and attribute database software (attribute tables). The data portal also provides a MODIS data extraction function that allows people to retrieve time series MODIS data (e.g., surface reflectance, vegetation indices) for individual pixels with geo-referenced field photos (Fig. 2a), which enables rapid analysis of phenology at individual POIs. With the contributions from citizen scientists and other researchers, the Field Photo Library now hosts over 30, 000 photos for Southeast Asia.

In this study, we first selected field photos that we collected during our field trips in Thailand, Vietnam and Indonesia; ∼7,000 geo-referenced field photos were collected in the past few years. Casio EX-H20 GBK Hybrid-GPS digital cameras were used in field trips to acquire GPS-referenced photos. All participants in the field trips used a standard protocol for taking photos in the field. For photos that describe high quality study sites, one takes photos from the center of the area of interest (one photo looking down and one photo looking up) and photos in each of four directions (N, E, W, and S) for a general description of the landscape, which results in a minimum 6 photos per site. For photos that describe only landscapes, photos were taken often from one or two directions (see the Field Photo Library website for detailed description of the field photo protocol). For the countries with limited field photos (Myanmar, Cambodia, Malaysia, and Philippines), ∼500 landscape photos with GPS information were collected from the Degree Confluence Project (confluence.org) and the Panoramio (www.panoramio.com). Together, a comprehensive coverage of geo-referenced field photos was achieved in all the countries except for Brunei, East Timor, and Singapore (Fig. 2a).

### Regions of Interest (ROIs) for algorithm training and product validation

We combined the geo-referenced field photos (POIs) and high-resolution images in Google Earth to generate homogeneous land cover polygons (polygon sampling units). The procedure is composed of two steps: (1) overlay geo-referenced field photos with high-resolution images in Google Earth; and (2) digitize high-resolution images in Google Earth to generate polygons. Previous studies have shown that Google Earth is feasible for ROI digitization of land cover classifications [56]–[59]. Our previous studies also showed that integrating the Field Photo Library and Google Earth is reliable [47], [60]. During the digitalization process, the minimum size of a polygon is required to be at least 3 times larger than the minimum land size used in the forest cover definition. For example, FAO provides a classic forest definition with three components: 1) tree canopy cover >10%, 2) tree height >5 m, and 3) minimum land size 0.5 ha [61]. We used this FAO definition of forest cover and ensured that each polygon has a size of at least 3 times larger than 0.5 ha and tree canopy cover >10%.

The resultant polygon sample units, called the regions of interest (ROIs), were then used for both algorithm training and product validation, respectively. A total of 78 ROIs for four land cover types (forest, cropland, water body, and built-up land) were generated as the algorithm training ROIs (Fig. 2b), and they were the same as those used in our previous study in mainland Southeast Asia [47], including 25 forest ROIs (997 986 pixels), 32 cropland ROIs (160 916 pixels), 10 water ROIs (303 948 pixels), and 11 built-up land ROIs (26 970 pixels). As the PALSAR L-band backscatter reflects more about physical characteristics, we assume the same algorithm will work in the insular Southeast Asia as well. The initial aim of this study is to verify whether the algorithm developed in the Mainland Southeast Asia [47] is suitable in insular Southeast Asia.

We also developed a second set of ROIs for validation of the results in this study (Fig. 2b). A total of 1,233 ROIs (11.2×10^5^ pixels) were collected, including 422 forest ROIs (458 488 pixels), 599 cropland ROIs (129 655 pixels), 70 water ROIs (445 538 pixels), and 142 other land cover ROIs (mainly built-up land, 88 019 pixels). The mean size of forest ROIs was bigger than those of cropland and built-up land, as the cropland and built-up landscapes are fragmented in Southeast Asia.

### Land cover classification based on PALSAR data and decision tree algorithm

Signature analyses of PALSAR backscatter values were conducted based on the ROIs for algorithm training. We calculated the mean and frequency histograms of backscatter values for the four land-cover types. These land cover types have distinguishable backscatter characteristics. Water has the lowest HH and HV backscatter due to its depolarization effect and strong absorption of energy. According to our definitions in this study, forest has a height over 5 m and has higher HH and HV backscatter due to more canopy backscatter in tree trunks and leaf canopy. Croplands have lower HH and HV backscatter than forest. Built-up lands show very complex characteristics as buildings are complex and building orientations and corner reflectors exist [47].

**Figure 3:**
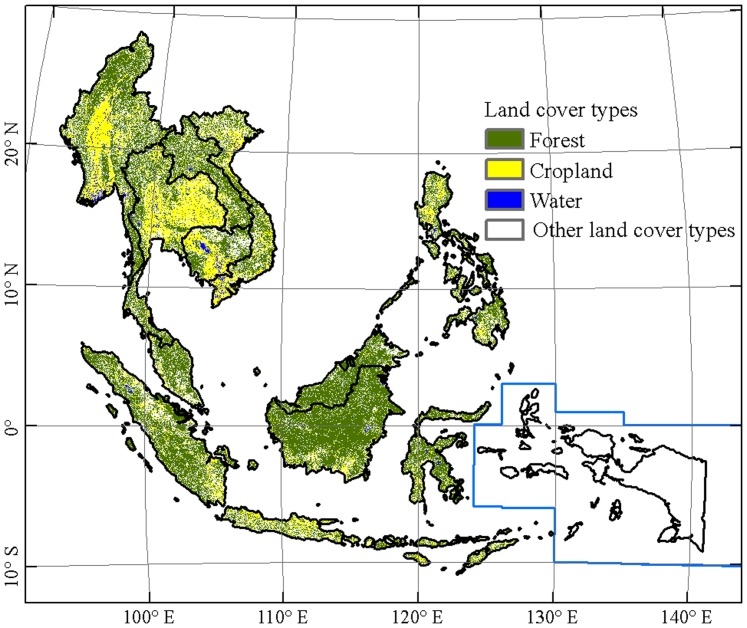
The resultant land cover map of Southeast Asia based on the PALSAR 50-m orthorectified mosaic data in 2009 and the decision tree algorithm.

The thresholds in decision tree rules were determined according to a statistical analysis of these ROIs. When we digitized the training ROIs, we tried to find the area with pure land covers, by assuming that the pixels within a 95% confidence interval of backscatter values are pure land cover types, and other pixels with the lowest and highest 2.5% backscatter values are likely mixed land cover types. The resultant decision tree rules and PALSAR data enable a simple and consistent approach to generate a continental scale forest cover map in Southeast Asia. For water body pixels, HH<−16 and HV<−24 threshold values were used. For forest pixels, we used 3.5<Difference image <6.5, −15<HV<−7 and 0.3<Ratio image<0.7 threshold values. For cropland or grassland pixels, we used HV<−16 threshold values. We then assigned the remaining pixels as other land covers including built-up land and shrubs, etc. A detailed description of the backscatter signature of land cover types was recently reported in our previous study [47]. The land cover map was generated by using the decision tree function in ENVI 5.0 software (Fig. 3), and then converted into a forest/non-forest map.

### Validation with ground truth data

A systematic and rigorous validation is always an essential concern in land cover classification, and is often carried out by comparing resultant maps with (a) a higher-resolution imagery or land cover product, and/or (b) ground truth samples from field trips. Field trip samples are the most straightforward validation references. Based on the validation ROIs mentioned in Section 2.4, validation of the PALSAR-based land cover map was conducted, and the confusion matrix was reported in Table 1, including user's accuracy, producer's accuracy, and overall accuracy.

**Table 1:** The confusion matrix between PALSAR-based land cover classification in Southeast Asia and Regions of Interest (ROIs) by referring to field photos and Google Earth.

	Class	Ground truth (GT) samples (pixels)				Total classified pixels	User Acc. (%)
		Forest	Cropland	Water	Others		
Classification	**Forest**	392800	2383	91	29036	424310	93%
	Cropland	9837	102247	10434	1001	123519	83%
	Water	25	18090	434735		452850	96%
	Others	55826	6935	278	57982	121021	48%
Total GT pixels		458488	129655	445538	88019		
Prod. Acc. (%)		86%	79%	98%	66%		

### Comparison between PALSAR-based forest map and other products

In addition to the above-mentioned validation with ground truth data, we also compared the PALSAR-based forest map with the two land cover products at three levels (national, subnational, and pixel levels): (1) GlobCover 2009 [21] and (2) MCD12Q1 2009 [19]. The FAO FRA 2010 statistics [8] were also used to do comparisons with the other three land cover products at the national level as it has no statistical data at subnational or smaller scales. Subnational boundary data were obtained from the GADM database of Global Administrative Areas (http://www.gadm.org/home). The pixel level comparison was conducted by aggregating these spatial forest datasets (PALSAR, MCD12Q1, and GlobCover) into forest fractional maps with 1.5-km×1.5-km resolution (1.5-km is the lowest common multiple of 50-m, 300-m, and 500-m).

As different land cover products have different classification schemes, we merged forest-related classes before the comparisons. GlobCover has 22 land cover types, and seven forest-dominated types were combined into one forest layer: closed to open broadleaved evergreen or semi-deciduous forest, closed broadleaved deciduous forest, open broadleaved deciduous forest/woodland, closed needle-leaved evergreen forest, open needle-leaved deciduous or evergreen forest, closed to open mixed broadleaved and needle-leaved forest, mosaic forest or shrubland/grassland. MCD12Q1 has several land cover classification schemes, and we used the classification scheme from the International Geosphere Biosphere Programme (IGBP) with 17 land cover types. Five forest types (evergreen needleleaf forest, evergreen broadleaf forest, deciduous needleleaf forest, deciduous broadleaf forest, and mixed forest) were combined into one forest layer. Land cover categories in FRA 2010 include forest, other wooded land, other land and inland water bodies [8]; only the forest category was used in this study. In addition, the GlobCover and MCD12Q1 datasets used different forest definitions. The GlobCover dataset uses 15% tree canopy cover and >5 m tree height, while the MCD12Q1 dataset uses 60% tree canopy cover and >2 m tree height [19], [21].

### Forest fragmentation analysis

Forest fragmentation has substantial impacts on animal and plant habitat quality and biodiversity [48], [49]. One forest fragmentation model was recently developed and it considers two indicators: forest area density (*P_f_*) and forest connectivity (*P_ff_*), within a certain “window” or “landscape” [48]. It has been widely used to assess the forest fragmentation by using various satellite-derived forest maps [62], [63]. We employed this forest fragmentation model to conduct a forest fragmentation analysis. The forest fragmentation was calculated based on a forest/non-forest binary map (forest = 1 and non-forest = 0). The two indicators were calculated with the following equations, 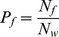
(2)

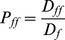
(3)where *P_f_* is the proportion of forest pixels in a certain window (e.g., 9×9), and is calculated by dividing forest pixels (*N_f_*) in a certain window by the total number of pixels (*N_w_*); *P_ff_* is the forest connectivity, and is calculated by dividing the pixel pair number that includes at least one forest pixel (*D_ff_*) by the pixel pair number that includes two forest pixels in cardinal directions (*D_f_*).

The resulting forest fragmentation map is grouped into six fragmentation categories. The fragmentation categories and their criteria are: (1) Patch, if *P_f_* <0.4; (2) Transitional, if 0.4<*P_f_* <0.6; (3) Perforated, if *P_f_*>0.6 and *P_f_* - *P_ff_* >0; (4) Undetermined, if *P_f_* >0.6 and *P_f_*  = *P_ff_*; (5) Edge, if *P_f_* >0.6 and *P_f_* <*P_ff_*; and (6) Interior, if *P_f_*  = 1.0 [48]. Using the 50-m PALSAR-based forest map in 2009 as input data, the model was applied to calculate forest fragmentation, under three window sizes: 9×9 pixels (450-m×450-m), 21×21 pixels (1050-m×1050-m), and 101×101 pixels (5050-m×5050-m).

## Results

### Forest map from the PALSAR 50-m mosaic imagery in 2009

The resultant PALSAR-based forest map in 2009 estimates a forest area of 2.56×10^6^ km^2^ for Southeast Asia (the forest area in the West Papua and Papua regions was filled using MODIS-based results due to PALSAR data unavailability, Table 2), accounting for more than half of the entire land area in the region. A large proportion of forests are concentrated in the northern hilly regions of mainland Southeast Asia, western Sumatra and the island of Borneo (Fig. 3). The land cover classification of four broad land cover categories from the PALSAR 50-m mosaic imagery has a high accuracy. The producer's accuracy and the user's accuracy of forest were 86% and 93%, respectively, which indicates that the accuracy of the PALSAR-based forest map at 50-m resolution was good and the PALSAR mosaic data performs well in continentally consistent forest mapping, which could be partly attributed to the simple classification scheme.

**Table 2:** National forest area comparison among three forest maps from PALSAR, GlobCover, MCD12Q1 in 2009 as well as the statistical data of FAO FRA 2010 (Unit: ×10^3^ km^2^).

	PALSAR	FAO FRA	GlobCover	MCD12Q1
Brunei	5	4	3	5
Myanmar	343	318	235	359
Cambodia	70	101	56	55
East Timor	7	7	1	2
Indonesia	885*)+419**)	944	860	1396
Laos	148	158	112	184
Malaysia	222	205	180	274
Philippines	140	77	82	153
Singapore	0	0	0	0
Vietnam	128	138	111	131
Thailand	190	190	93	128
Total areas	2556	2141	1733	2688

*) Excluding the West Papua and Papua regions (including Maluku and North Maluku) due to the original data missing of PALSAR 50 m Orthorectified mosaic product in 2009.

**) The area of the West Papua and Papua regions was complemented according to the MODIS land cover (MCD12Q1) product.

### Comparison of the forest products from PALSAR, MERIS, MODIS, and FRA

**Figure 4:**
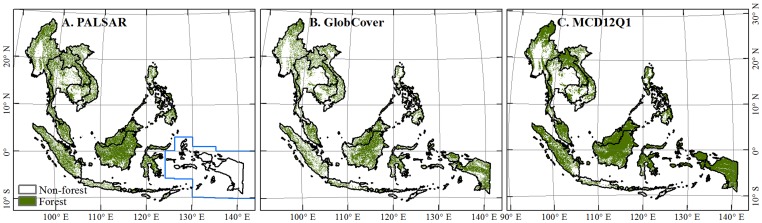
The spatial distribution of forest cover in the Southeast Asia from (*A*) PALSAR 2009 forest map, (*B*) GlobCover 2009 forest map, and (*C*) MCD12Q1 2009 forest map. The blue box in *A* shows the region missing PALSAR data (the West Papua and Papua regions).

At the continental level, the estimate of forest area from the PALSAR-based forest map (2.56×10^6^ km^2^, Table 2) is very close to the MCD12Q1 2009 (2.69×10^6^ km^2^), and higher than the FRA 2010 (2.14×10^6^ km^2^), and the GlobCover 2009 (1.73×10^6^ km^2^). The differences in forest area estimates between the PALSAR-forest map and that from GlobCover and FRA are significant. All the three products from PALSAR, MODIS and MERIS show generally consistent spatial distributions of forest but differ in some regions (Fig. 4). Different spatial resolutions of these three maps show varying visual effects.

At the national level, the forest area estimates from the four datasets were compared and they differed in most countries (Table 2). PALSAR-based forest estimates are more consistent with those of MCD12Q1 at national scale. Root-mean-square deviation (RMSD) of national forest areas is 111×10^3^ km^2^ between PALSAR and FRA, 143×10^3^ km^2^ between PALSAR and GlobCover, and 39×10^3^ km^2^ between PALSAR and MCD12Q1, respectively. In mainland Southeast Asia, the PALSAR-based forest estimates are closer to FRA with RMSD 19×10^3^ km^2^, but differ slightly from GlobCover and MCD12Q1 with RMSD of 68 and 34×10^3^ km^2^, respectively, which is consistent with our previous study [47]. The forest area estimates varied substantially in Indonesia and the Philippines. In Indonesia, PALSAR and MCD12Q1 estimate 1304 and 1396×10^3^ km^2^ forest respectively but the FAO FRA and GlobCover estimate 860 and 944×10^3^ km^2^ forest respectively. In the Philippines, PALSAR and MCD12Q1 estimate 140 and 153×10^3^ km^2^ forest respectively but the FAO FRA and GlobCover estimate 77 and 82×10^3^ km^2^ forest respectively. Even with the large differences in some countries, the correlation coefficients between PALSAR-based forest areas and those from the other three sources were all significant at a national level (*P*<0.001, n = 11, see Table 2).

**Figure 5:**
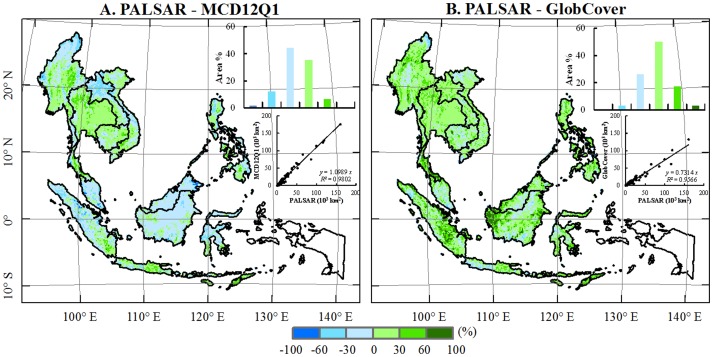
The comparison between the three fractional forest maps at a spatial resolution of 1.5-km by 1.5-km gridcell: (*A*) PALSAR – MCD12Q1 and (*B*) PALSAR – GlobCover. The maps show the differences between two maps. The inset histograms show frequencies at various levels of difference between two maps. The scatter plots show the comparison in forest area at the sub-national level among PALSAR, MCD12Q1 and GlobCover in 2009. The data from the four provinces (Irian Jaya Barat, Maluku, Maluku Utara, and Papua) are excluded due to missing PALSAR data.

At the subnational (i.e., provincial) level, the forest areas from PALSAR, MCD12Q1, and GlobCover products were compared. Due to the PALSAR data missing in the West Papua and Papua region, we excluded the four provinces of Irian Jaya Barat, Maluku, Maluku Utara, and Papua from data analysis, and 269 subnational regions were used for the comparison. The PALSAR-based forest areas are significantly correlated with the MCD12Q1 forest areas (*y* = 1.0989*x*; *R* ^2^ = 0.98, *p*<0.0001, *n* = 269), and are approximately 10% lower than the MCD12Q1-based forest estimates (Fig. 5A). PALSAR-based forest areas are also linearly correlated with the GlobCover forest areas (*y* = 0.7314*x*, *R* ^2^ = 0.96, *p*<0.0001, *n* = 269), but are 27% higher than the GlobCover forest estimates (Fig. 5B).

At the pixel level, we compared these three datasets (PALSAR, MCD12Q1, GlobCover) as aggregated grids. We first defined a common size of grid cell (1.5-km×1.5-km) for the three forest maps and calculated percentages of forest areas within individual grid cells. We then calculated the differences between two fractional forest maps (PALSAR vs. MCD12Q1; PALSAR vs. GlobCover). For the PALSAR and MCD12Q1 pair, approximately 79% of grid cells fell within +/− 30% discrepancies, distributed mostly throughout Thailand, Borneo, and Cambodia (Fig. 5A). In those areas with positive differences (>30%) (e.g., the central plain of Thailand and Java Island), the PALSAR map identified more forest areas than the MCD12Q1 map, where there are extensive croplands and cities but small patches of forests. This is attributed to the fact that PALSAR 50-m mosaic imagery can identify small patches of forests, but moderate spatial resolution MODIS (500-m) and MERIS (300-m) images tend to miss them. In those areas where the PALSAR identifies less forest areas than the MCD12Q1 and GlobCover, there are large areas of forests, for example, northern Myanmar, central Borneo, northern Sumatra and the central Sulawesi Islands. That is because 50-m PALSAR images can identify small gaps or non-forest patches while MODIS (500-m) and MERIS (300-m) images cannot identify them. For the PALSAR and GlobCover pair comparison, approximately 76% of grid cells fall within +/−30 discrepancies. The mild positive differences (0–30%) between PALSAR and GlobCover datasets occur in 50% of the grid cells of the study area. In the west of Borneo Island, south of Thailand, and mid-eastern area of Sumatra Island, the PALSAR-based fractional forest map is about 60% to 100% higher than that of the GlobCover dataset (Fig. 5B), where tree plantations (e.g., rubber plantation) were widely distributed.

### Forest fragmentation assessment from the PALSAR forest map at 50-m resolution

**Figure 6:**
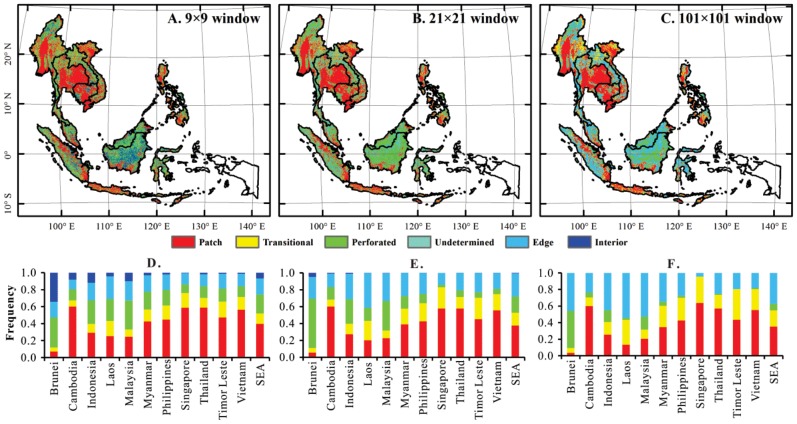
The spatial distribution of forest fragmentation in Southeast Asia with (*A*) 9×9 pixel window, (*B*) 21×21 pixel window, and (*C*) 101×101 pixel window. The stacked-bar histogram charts (*D*, *E*, and *F*) under the maps show the percent areas of five forest fragmentation categories corresponding with the forest fragmentation maps (*A*, *B*, and *C*).

**Figure 7:**
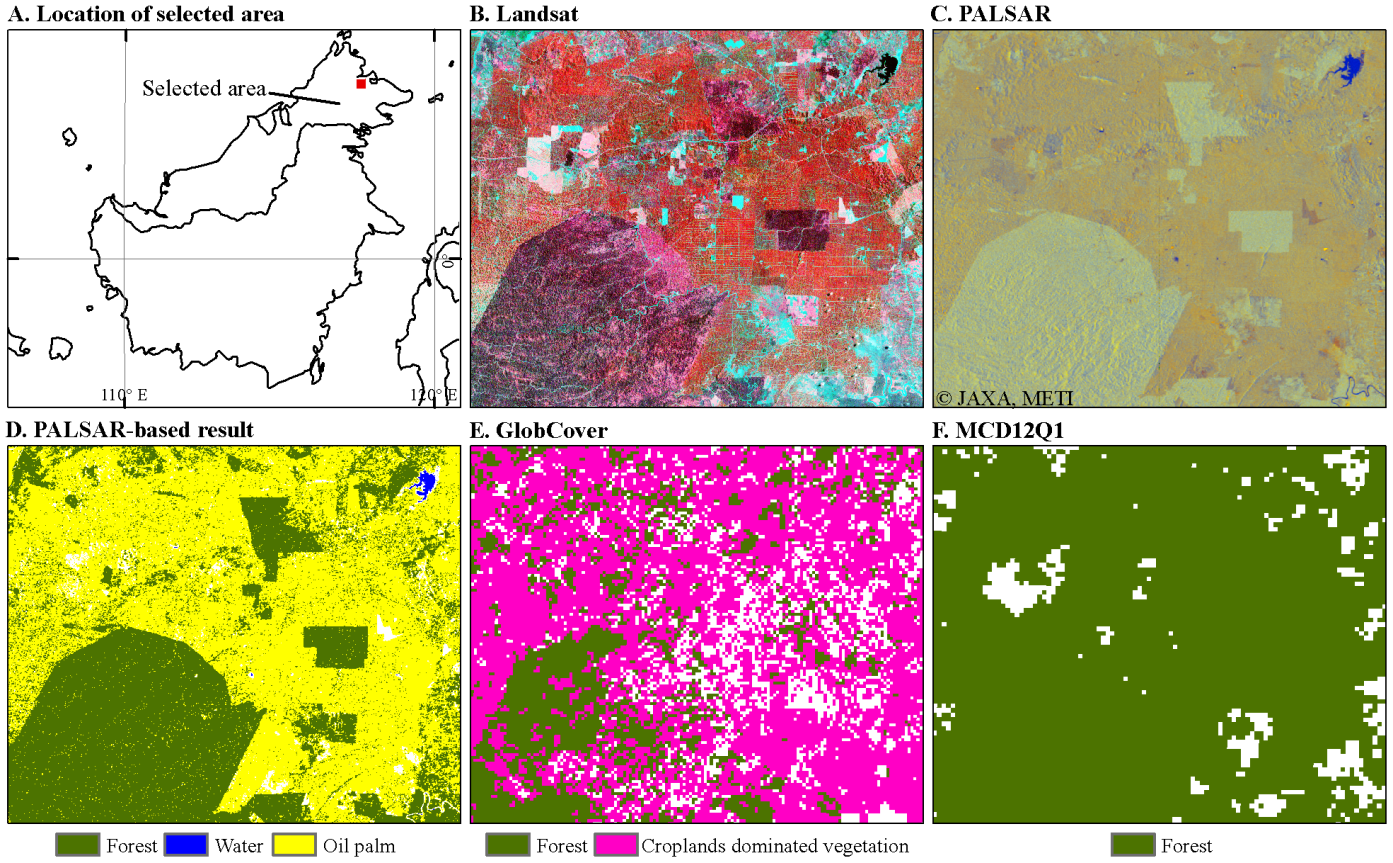
Visual interpretation and comparison of different land cover products in a region mixed with natural forest and oil palm plantation. A) the location of the case region in Borneo Island, Southeast Asia; B) the false color composited graph of Landsat 5 image (30 m, path/row = 117/56, R/G/B  =  Band NIR/Red/Green) on August 11, 2009; C) the false color composited graph of PALSAR image (R/G/B  =  HH, HV, HH/HV) in 2009; D) PALSAR-based land cover map (50 m) in 2009 from this study; E) GlobCover 2009 land cover map (300 m); and F) MCD12Q1 2009 land cover map (500 m). The differences of these three products in separating natural forest and oil palm plantation are obvious, MCD12Q1 considers oil palm plantation as forest, while GlobCover and PALSAR don't, and PALSAR has better performance in separating natural forest and oil palm plantation.

We used the fragmentation model mentioned in Section 2.7 and the PALSAR-based forest map to calculate forest fragmentation under three window sizes. The “patch” area percentage, which represents the highest level of forest fragmentation, is estimated to be approximately 39.7% in the fragmentation index map with a 9×9 window (450-m×450-m), 37.7% in the map with a 21×21 window (1050-m×1050-m), and 35.5% in the map with a 101×101 window (5050-m×5050-m) (Fig. 6). Most of the “patch” area is distributed in those regions dominated by urban and croplands (Figs. 7*A*-*C*). The “interior” area, which represents the lowest level of forest fragmentation or intact forest, is estimated to be approximately 6.6% in the fragmentation index map with 9×9 pixels, 0.4% in the map with 21×21 pixels, and 0% in the map with 101×101 pixels (Fig. 6). Most of the “interior” area is distributed on Borneo Island. The lowest (interior) and highest (patch) levels of forest fragmentation tend to decrease in relation to an increase in window size (Fig. 6). Thus, the fragmentation index is spatial scale-dependent. The large window size (e.g., 101×101 pixels in this study) could reduce the estimates of both “patch” and “interior” areas. The finer resolution (e.g., 50-m PALSAR) forest map enables us to carry out forest fragmentation analysis at a small window size (e.g., <1000-m) and to quantify forest fragmentation with higher accuracy and finer details. Accurate fragmentation information from the 50-m forest cover map in this study could better serve the scientific and management communities for biodiversity conservation.

## Discussion

### Advantages of PALSAR-based tropical forest mapping

PALSAR-based tropical forest mapping in this study has three advantages: a) cloud-free capability, b) high spatial resolution, and c) a simple algorithm that is built upon the strength of radar data which are calibrated physical measurements. First, cloud-free land observation capability improved data accessibility (complete spatial coverage) which is the largest obstacle or source of uncertainty for land cover mapping in the moist tropical regions [64], [65]. Second, the 50-m forest map is the first forest map in Southeast Asia with a finer resolution than one hundred meters [4], [22], which is especially meaningful for Southeast Asia as it has fragmented landscapes in most areas. Previous optical images with hundred-meter resolution had difficulty in acquiring such detailed information in complex landscapes. Third, several studies used the unsupervised classification method for forest mapping in Southeast Asia, due to the spatial heterogeneity present in the large extent [4], [15]–[18], [22]. However, the unsupervised method is time consuming and requires experts with local knowledge for post-classification interpretation and labeling. In this study, we developed and used a continentally consistent decision tree algorithm to map forest in Southeast Asia with a great time savings and limited human and financial resources. When using the PALSAR-based forest map as a reference map, which has finer spatial resolution and higher accuracy in forest mapping, our analysis suggests that the MCD12Q1 data product could overestimate forest areas while the GlobCover data product is likely to underestimate forest areas in the region.

### Sources of uncertainty and discrepancy among the forest maps

Several sources of uncertainty exist for PALSAR-based mapping of tropical forests, and they include relief-effects, seasonal effects, spatial resolution of imagery, forest definition, and accuracy assessment. The relief-effect is always a challenge to tackle with SAR data [43], [66], which tends to underestimate forest areas in the mountainous regions such as northern IndoChina, western Sumatra and Borneo. However, Figure 5 shows that PALSAR did not significantly underestimate forest in those regions when compared with the GlobCover and MCD12Q1 datasets; and limited underestimates might exist in the border regions of northern Myanmar and northern Vietnam (Fig. 5). Therefore, the effect of relief on the resultant forest map must exist but could be limited. The PALSAR images were acquired from June to October in 2009, which is part of the wet season in some areas; and water content of soils in the wet season could decrease the backscatter of forests and cause the underestimate of forest areas to some degree.

Scale and resolution issues greatly affect the forest definitions and resultant forest cover maps. Different spatial resolutions of satellite images have different representation capabilities against the definition of forest cover. The pixel sizes in the GlobCover and MCD12Q1 datasets are much larger than the minimum unit area (0.5 ha) of FAO forest definition, and they tend to have more mixed pixels. Therefore, forest maps derived from finer resolution image data tend to reduce these uncertainties in forest area estimations. In this study, the 50-m spatial resolution PALSAR imagery reduces the issue of mixed pixels substantially, which has led to higher accuracy forest cover maps, when compared to 300-m (MERIS) and 500-m (MODIS) imagery.

In addition, the discrepancies among these four datasets could be in part attributed to the aggregation processes of the MCD12Q1 and GlobCover forest-related land cover types in this study, as they use different land cover classification schemes [19], [21], [61]. In the GlobCover 2009 land classification system, several land cover classes are a mixture of multiple vegetation types due to the coarse spatial resolution, e.g. the type of “mosaic vegetation (grassland/shrubland/forest)/cropland.” In the FAO FRA 2010 report, forest areas excluded those with agricultural purposes or urban development. However, it is difficult to distinguish detailed forest use types (forestry use or agricultural use targets) in the remote sensing datasets at moderate spatial resolutions. One reason for their area discrepancy (Table 2) may be a large area of plantations, since they are considered forest in some products such as the PALSAR-based forest in this study and MCD12Q1, while it is not in the FAO FRA and GlobCover. For example, according to the FAO FRA 2010 report, Indonesia and the Philippines have a higher “Other wooded land” proportion (2.1×10^5^ km^2^ and 1.0×10^5^ km^2^ respectively) [8].

The accuracy assessment of forest cover maps at continental and global scales is a very challenging task, as few research projects are able to carry out a rigorous sampling design (e.g., a systematic sampling) [67], [68] due to limited budgets and human resources. In our project we were also unable to carry out statistically rigorous sampling design for our field trips. In this study, the accuracy assessment of our PALSAR-based forest map depends on the availability of geo-referenced field photos from the Global Geo-Referenced Field Photo Library, which is a community remote sensing and citizen science data portal. The long-term objective of the Field Photo Library is to help develop a systematic sampling design with geo-referenced field photos available in each sampling grid. At this moment, the Field Photo Library is still in an early stage, and it does not have field photos in many large areas (e.g., Borneo Island and Maylasia, see Fig. 2), despite a collection of 30 000+ photos in Southeast Asia. As this study and previous studies [47], [69] demonstrate the value of geo-referenced field photos as ground truth data, more contributions from the scientific community and citizen scientists are likely to occur in the near future. Recently we have released “Field Photo” iPhone App and it is freely available to the public, which may encourage more stakeholders and citizen scientists to take geo-referenced field photos and share them. The application can be downloaded at iTunes. We believe that the Field Photo Library will grow substantially and rapidly, and provide an effective means of in-situ data collection that could meet the needs for systematic sampling design in regional or global land cover accuracy assessment in the future.

### An issue on separability between natural forests and forest plantations

Several land cover classification maps that used optical images (e.g., MCD12Q1) do not separate natural forests from forest plantations due to their similar spectral characteristics [19], [21]. Here we used natural rubber and oil palm, two important plantations widely distributed in Southeast Asia, as preliminary examples to evaluate to what degree these four data products separate natural forests and forest plantations.

Southeast Asia contains about 75% the world's rubber plantations (1.01×10^5^ km^2^), according to an incomplete statistic from the FAO FRA 2010 [8]. Rubber plantations were included as forest in the FRA report [8]. Rubber plantations were also included in our PALSAR-based forest map as well as the MODIS and MERIS-based forest maps, as verified according to the ground truth data of rubber plantations from the Field Photo Library. That is, the spectral or backscatter based remote sensing approaches used in these three products did not separate rubber plantations from natural forests. Previous studies have shown that rubber plantations and natural forests have similar physical and spectral characteristics during most parts of the plant growing season, when optical remote sensing images were used [70]. Our previous studies indicated that deciduous rubber plantations can be identified by integrating phenological information from time series optical imagery (e.g., MODIS or Landsat) and forest structure information from PALSAR [60], [69].

Oil palm is another important plantation type in Southeast Asia. Indonesia and Malaysia are the largest oil palm producers in the world, and their production of palm oil accounts for 87% of the global production [71]. In oil palm plantations, oil palm trees are planted in unique spatial structures. Fig. 7 shows the forest maps from different products in a sample region of Borneo Island, where oil palm plantations and natural forests are mixed together (Fig. 7A). By referring to the 30-m Landsat color composition map in 2009 (Fig. 7B), we see that PALSAR has better performance in separating natural forests and oil palm plantations than the GlobCover and MCD12Q1 datasets. The MCD12Q1 does not separate oil palm plantations and natural forests, and reports only the forest category (Fig. 7F). The GlobCover considers oil palm plantations as cropland or a mosaic of cropland and vegetation (Fig. 7E). PALSAR also has higher spatial resolution than GlobCover and it can show a clear boundary between natural forests and oil palm plantations (Fig. 7C, D & E). The specific physical shape (large crown and less branches) of the oil palm trees [72] causes higher HH and lower HV backscatter, resulting in the larger difference between HH and HV. Two recent studies used the backscatter difference between HH and HV as the primary indicator to map oil palm plantation, and pixels with HH-HV>6.5 are identified as oil-palm plantations [10], [53]. The HH-HV<6.5 rule was used in the decision tree algorithm in this study and our previous study [47], and the PALSAR-based forest map does not include oil palm plantations. The rapid expansion of oil palm plantations in Southeast Asia calls for an enhanced capability for mapping oil palm plantations and natural forests.

### Potential applications of the PALSAR 50-m forest map in forest fragmentation

We showcased the application of the 50-m forest map for analysis of forest fragmentation, which plays an important role in ecosystem services and biodiversity. The analysis of forest fragmentation was found to be dependent on the spatial scale (window size), which is consistent with previous studies [13], [73], for example, Figure 6 indicates that there is much less interior forest area when using a 101×101 window than using a 9×9 window. The spatial comparisons of fragmentation at national levels are meaningful only with consistent window size. The smaller window tends to give more details about the forest fragmentation pattern; also, the accuracy of the original forest map plays a critical role in the accurate evaluation of forest fragmentation. Our 50-m forest map in this study has richer spatial information than the existing optical remote sensing products and is expected to provide more effective support for evaluation of forest fragmentation. By comparing the fragmentation map (Figure 6) with a potential species richness projection from a previous study [74], we found that forest fragmentation was spatially consistent with the potential species richness. Consequently, the forest fragmentation map could contribute to the biodiversity conservation and habitat planning.

The fragmentation map is also expected to provide decision support for the forest management. The fragmentation level of the interior is mainly located in Borneo Island according to Figure 6a, and is surrounded with some pixels with edge and perforated classes, which implied that the primary tropical forest has been deforested into a fair number of small holes. That is also shown in Figure 6b, where the same regions showed as edge classes at the 1050-m window. The land use conversion to plantations was also reported in previous studies [75], [76]. The areas with less severe edge effects and may recover more easily and convert to interior forest.

The resultant 50-m forest cover map in this study is also expected to serve as a baseline map for forest resource inventory, reducing emissions from deforestation and forest degradation (REDD+) implementation, deforestation monitoring, biodiversity conservation, and habitat planning in Southeast Asia. For example, previous studies have shown that the largest source of uncertainties in estimates of carbon emissions from deforestation in the tropical zone is attributed to the poor quality of available deforestation maps [29], [30], [77]. This highly accurate forest map will support carbon emission estimation more effectively. Also, the continuous L-band SAR data will facilitate forest change detection in the long term. Although PALSAR was out of service as of April 2011, the Advanced Land Observing Satellite-2 (ALOS-2) will succeed the mission with enhanced capabilities. By integrating forest maps from JERS-1 images, forest changes from the 1990s to circa 2015 can be quantified in the foreseeable future [45].

## Conclusion

Tropical forests play an important role in the carbon cycle, biodiversity conservation, and other ecosystem services closely related to human well-being. Our understanding of these environmental issues is dependent on the resolution and accuracy of the tropical forest maps. For example, the applicability and reliability of forest fragmentation evaluation greatly depends on the spatial resolution of forest maps. To our knowledge, this is the first 50-m forest cover map of Southeast Asia, derived from the cloud-free PALSAR 50-m orthorectified mosaic imagery. Our preliminary analysis highlights the potential contribution of this 50-m forest cover map to forest fragmentation analysis. The PALSAR-based forest map can be anticipated to serve in forest resource inventory, deforestation monitoring, REDD+ implementation, and biodiversity conservation, and it can also be used to improve simulations of regional and global biogeochemical, hydrological, and climate models [28].
